# ViralConsensus: a fast and memory-efficient tool for calling viral consensus genome sequences directly from read alignment data

**DOI:** 10.1093/bioinformatics/btad317

**Published:** 2023-05-12

**Authors:** Niema Moshiri

**Affiliations:** Department of Computer Science & Engineering, UC San Diego, La Jolla, CA 92093, United States

## Abstract

**Motivation:**

In viral molecular epidemiology, reconstruction of consensus genomes from sequence data is critical for tracking mutations and variants of concern. However, as the number of samples that are sequenced grows rapidly, compute resources needed to reconstruct consensus genomes can become prohibitively large.

**Results:**

ViralConsensus is a fast and memory-efficient tool for calling viral consensus genome sequences directly from read alignment data. ViralConsensus is orders of magnitude faster and more memory-efficient than existing methods. Further, unlike existing methods, ViralConsensus can pipe data directly from a read mapper via standard input and performs viral consensus calling on-the-fly, making it an ideal tool for viral sequencing pipelines.

**Availability and implementation:**

ViralConsensus is freely available at https://github.com/niemasd/ViralConsensus as an open-source software project.

## 1 Introduction

Viral molecular surveillance, a technique in which viral genomes are reconstructed from sequence data generated from samples collected from patients as well as the environment (e.g. wastewater) and are monitored in real-time or near real-time, has been critical throughout the Coronavirus Disease 2019 (COVID-19) pandemic (Oude Munnink et al. 2021; Karthikeyan et al. 2022). The reconstruction of consensus genome sequences from raw sequence data requires the use of various bioinformatics pipelines such as CoVpipe (https://gitlab.com/RKIBioinformaticsPipelines/ncov_minipipe), CoVpipe2 (https://github.com/rki-mf1/CoVpipe2), HAVoC (Truong Nguyen et al. 2021), V-pipe (Posada-Céspedes et al. 2021), ViReflow (Moshiri et al. 2022), and many others, which can be slow and can require non-trivial computational expertise.

The current best-practice pipeline for reconstructing a consensus genome sequence from raw viral amplicon sequence data is the iVar pipeline (Grubaugh et al. 2019). First, reads are mapped to the reference genome using a read mapper such as Minimap2 (Li 2018) or BWA (Li and Durbin 2009) and position-sorted using Samtools (Li et al. 2009). Next, reads are primer- and quality-trimmed using iVar and again position-sorted using Samtools. A pile-up is then computed from the sorted trimmed reads using Samtools, and a consensus genome sequence is called from the pile-up file using iVar. This position-sorted pile-up-based approach, which is either explicitly or implicitly utilized by all aforementioned consensus pipelines, is ideal for long genomes (e.g. human) in which the memory needed to store base counters for every position of the genome simultaneously would become prohibitively large, but due to their small length, viral consensus genome sequences can be computed much faster.

Here, we introduce ViralConsensus, a fast and memory-efficient tool for calling viral consensus genome sequences directly from read alignment data. ViralConsensus is orders of magnitude faster and more memory-efficient than existing methods. Further, unlike existing methods, ViralConsensus can pipe data directly from a read mapper via standard input and performs viral consensus calling on-the-fly, making it an ideal tool for viral sequencing pipelines.

## 2 Results and discussion

ViralConsensus is a command-line tool written in C++ and depends on htslib (Bonfield et al. 2021). ViralConsensus takes the following as required input: (i) a SAM/BAM/CRAM file containing the mapped reads (or “-” to read from standard input), (ii) a FASTA file containing the reference genome, and (iii) an output FASTA file to write the consensus genome (or “-” to write to standard output). Optionally, the user can also provide the following: (i) an output file in which to write base counts at each position (or “-” to write to standard output), (ii) an output file in which to write the insertion counts (or “-” to write to standard output), (iii) a minimum quality threshold to count a base in a read (default: 20), (iv) a minimum depth threshold to call a position in the consensus (default: 10), (v) a minimum frequency threshold to call a base/insertion in the consensus (default: 0.5), (vi) a symbol to use for ambiguous positions that fail to meet the minimum depth or frequency thresholds (default: “N”), (vii) a BED file containing primer positions to trim (default: no primer trimming), and (viii) a number of positions beyond the end of a primer to also trim (default: 0).

First, the reference genome is loaded from file, and base/insertion counters for each position of the genome are preallocated. Then, if the user wishes to optionally primer-trim the reads (e.g. to avoid amplicon sequencing primers from biasing the consensus sequence toward the reference genome if the reads have not already been primer-trimmed prior to running ViralConsensus), the amplicon primer start and end positions are loaded from file, and for each position of the reference genome, the end position of the primer that spans that position (if any) is precomputed. Next, read alignments are streamed on-the-fly (without any need for sorting) and, for each column of a given alignment, if the user-provided base quality threshold is met, the base/insertion count at the corresponding position of the reference genome is incremented (with any positions covered by an amplicon sequencing primer skipped entirely). After all read alignments have been processed, a consensus sequence is constructed by iterating over the positions of the reference genome and outputting the most frequent base or insertion at any given position (or the user-provided ambiguous symbol if the user-provided minimum depth or base frequency thresholds are not met).

ViralConsensus is able to trim reads on-the-fly using the user-provided minimum base quality threshold (for quality-trimming) and primer file (for primer-trimming), but users can trim reads prior to executing ViralConsensus if desired. Because it performs all computations on-the-fly and does not require intermediary files, ViralConsensus can be easily integrated into existing pipelines by piping directly from the read mapper, significantly reducing disk I/O. Further, because of its approach, ViralConsensus has constant memory consumption and linear runtime with respect to sequencing depth, and it has linear memory consumption with respect to genome length.

In order to benchmark ViralConsensus with respect to sequencing depth, we obtained a Severe Acute Respiratory Syndrome Coronavirus 2 (SARS-CoV-2) amplicon sequencing dataset in which 2607 samples were sequenced PE150 across four lanes of an S4 flow cell to an average read count of 4.58 M read pairs per sample using the SWIFT v2 protocol on an Illumina NovaSeq 6000 (Moshiri et al. 2022). Samples were mapped to the NC_045512.2 reference genome using Minimap2. We selected the single highest-depth sample and randomly subsampled it to *n* = 100, 1K, 10K, 100K, and 1M successfully mapped reads, with 10 replicates for each *n*. We then ran ViralConsensus v0.0.1 as well as the iVar pipeline (Samtools v1.16.1 and iVar v1.3.1) to compute consensus sequences from each subsampled replicate.

**Figure 1.**
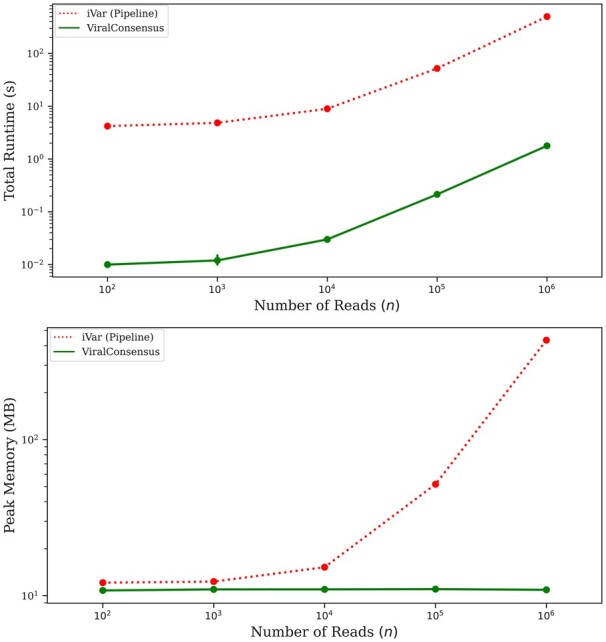
Performance benchmark. Total runtime (top) and peak memory (bottom) for SARS-CoV-2 sequence datasets with *n *=* *100, 1K, 10K, 100K, and 1M mapped reads. All runs were executed sequentially on a 2.8 GHz Intel i7-1165G7 CPU with 16 GB of memory.

As can be seen in Fig. 1, ViralConsensus is orders of magnitude faster and more memory efficient than the iVar pipeline, and it is able to call a consensus sequence from an amplicon sequencing dataset with 1 million reads in <2 s with a peak memory usage of <12 MB. Importantly, while both methods’ runtimes scale linearly with sequencing depth, the iVar pipeline’s peak memory usage grows substantially as sequencing depth increases, whereas the peak memory of ViralConsensus remains constant.

In order to assess the accuracy of the consensus sequences produced by ViralConsensus, we selected representative complete genome sequences from multiple SARS-CoV-2 lineages of interest (one sequence per lineage) from the NCBI Virus database, listed as Lineage (GenBankID) pairs: B (NC_045512.2), B.1.1.7 (LC650844), BA.1 (OQ523614.1), BA.2 (OQ194009.1), and XBB.1 (OQ346068.1). These specific lineages were selected to provide a range of representative substitutions, insertions, and deletions with respect to the NC_045512.2 reference genome that occur in real-world SARS-CoV-2 genome sequences, and these specific sequences were selected by filtering NCBI Virus for complete genomes with 0 ambiguous characters obtained from a human host with an isolation source of oronasopharynx. We then used ART version MountRainier-2016-06-05 to simulate Illumina HiSeq 2000 single-end short reads (Huang et al. 2012) and NanoSim-H v1.1.0.4 to simulate Oxford Nanopore Technologies (ONT) long reads (Yang et al. 2017; Břinda and Yang 2021) from each lineage genome, both using their default settings. We simulated datasets at 30×, 40×, and 50× coverage, and we simulated 10 technical replicates per lineage per sequencing technology per coverage. We then mapped the simulated reads to the NC_045512.2 reference genome using Minimap2’s short read (“sr”) and ONT (“map-ont”) presets, and we lastly reconstructed consensus sequences using the default settings of ViralConsensus v0.0.1 and iVar v1.3.1. To assess accuracy, each reconstructed consensus sequence was pairwise-aligned against its respective true sequence using MAFFT v7.505 (Katoh et al. 2002), and Hamming distances (total number of mismatches, insertions, deletions, and ambiguous symbols, normalized by alignment length) were computed from each pairwise alignment as a measure of consensus sequence error.

**Figure 2.**
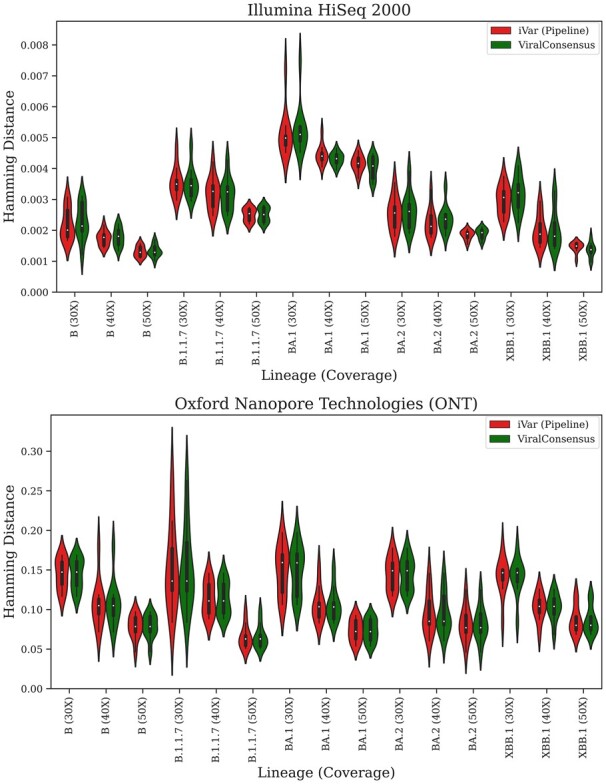
Accuracy benchmark. Hamming distance of reconstructed SARS-CoV-2 consensus sequences versus true sequences using simulated Illumina HiSeq 2000 (top) and Oxford Nanopore Technologies (bottom) reads.

As can be seen in Fig. 2, ViralConsensus produces consensus sequences that are as accurate as the iVar pipeline, and this observation was consistent across sequencing technologies, SARS-CoV-2 lineages, and sequencing coverage. Further, as expected, consensus sequence reconstruction error decreases as sequencing coverage increases. Interestingly (though unsurprisingly), reconstruction from ONT reads had higher error rates than reconstruction from Illumina reads at the same coverage. Unexpectedly, while consensus sequence reconstruction error was at similarly low levels across SARS-CoV-2 lineages, reconstruction of the BA.1 genome sequence from Illumina reads resulted in noticeably higher error rates than reconstruction of other lineages from Illumina reads. It is unclear if this increase in reconstruction error is attributed to features of the BA.1 lineage as a whole versus issues with the specific sequence that was chosen in this accuracy experiment, though thorough exploration of this phenomenon, as well as an exploration of the impact of different read mappers and mapping settings, is outside of the scope of this work and would be an interesting future research direction.

In its current form, ViralConsensus only takes into account base quality scores, not mapping quality scores. Further, in its current form, ViralConsensus only outputs a general ambiguous symbol (“N” by default) rather than more specific non-N ambiguous International Union of Pure and Applied Chemistry (IUPAC) symbols (e.g. “R” = “G or A”). However, support for these additional nuances can be implemented in future versions of ViralConsensus if requested by users. Further, while ViralConsensus was designed with short linear viral genome sequences in mind, it can be applied to consensus sequence reconstruction of other short linear non-viral genome sequences (e.g. mitochondrial DNA).

In sum, we introduce ViralConsensus, a fast and memory-efficient tool for calling viral consensus genome sequences directly from read alignment data. ViralConsensus is orders of magnitude faster and more memory-efficient than existing methods, yet it achieves similar accuracy. We hope ViralConsensus will aid viral molecular epidemiologists in their efforts to analyze viral sequence data at massive scale.

## Data Availability

All code and data are accessible at https://github.com/niemasd/ViralConsensus and https://github.com/niemasd/ViralConsensus-Paper.
